# Lipid-Derived Aldehydes: New Key Mediators of Plant Growth and Stress Responses

**DOI:** 10.3390/biology11111590

**Published:** 2022-10-29

**Authors:** Xin Liang, Ruyi Qian, Dan Wang, Lijuan Liu, Chengliang Sun, Xianyong Lin

**Affiliations:** 1 MOE Key Laboratory of Environment Remediation and Ecological Health, College of Environmental & Resource Sciences, Zhejiang University, Hangzhou 310058, China; 2 Iterdisciplinary Research Academy, Zhejiang Shuren University, Hangzhou 310015, China

**Keywords:** aldehydes, lipid, abiotic stress, detrimental effects, detoxification mechanisms, signaling effects

## Abstract

**Simple Summary:**

Aldehydes are inevitably produced through non-enzymatic and enzymatic pathways from fatty acids in both normal and stressed conditions. Different fatty acids result in unique aldehydes with carbon chains of different lengths and levels of unsaturation, which determine their functions and reactivities in plants. The detailed description of the formation, toxic effects, and detoxification mechanisms of aldehydes in plants is highlighted in this review. Moreover, the signaling effects of aldehydes are summarized as well.

**Abstract:**

Aldehydes, derivatives of lipids, are ubiquitously produced through non-enzymatic and enzymatic pathways in higher plants and participate in many physiological and biological processes. Increasing evidence demonstrates that aldehydes are involved in plants response to many abiotic stresses, such as light, drought, heat and nutrient deficiency. In plant cells, endogenously triggered or exogenously applied high concentrations of aldehydes can damage proteins and nucleic acid, disturb redox homeostasis, and consequently inhibit plant growth; therefore, they are considered cytotoxins. Aldehyde levels are also used as biomarkers to evaluate the health status of plants. Further genetic research shows that several enzymes have strong capacities to detoxify these electrophilic aldehydes. Small molecules, such as carnosine and glutathione, also exhibit the ability to scavenge aldehydes, effectively promoting plant growth. Recently, increasing evidence has shown that certain aldehydes at certain concentrations can upregulate survival genes, activate antioxidant responses, increase defense against pathogens and stimulate plant growth. This review summarizes recent studies of lipid-derived aldehydes in higher plants, mainly focusing on the generation pathway, toxic effects, and detoxification strategies. In addition, the signaling effects of aldehydes in plants are also discussed.

## 1. Introduction

**Figure 1:**
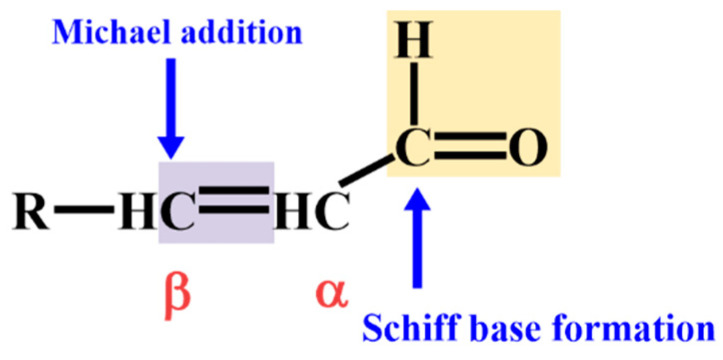
The structure of *α*,*β*-unsaturated aldehydes. The aldehyde group and C-C double bond indicated in red are mainly responsible for Schiff base formation and Michael addition, respectively. R represents a hydrocarbon group(s) or a hydrogen atom. Modified from Saxena et al. [11].

Aldehydes, a large class of electrophilic compounds bearing aldehyde groups (-CHO), are inevitably and consistently produced during cell metabolism and growth in various living organisms [1,2]. At present, it is well-known that aldehydes are derived from lipid peroxides (LOOH) through the action of radical oxidants, especially polyunsaturated fatty acid (PUFA) peroxides [3,4,5]. Plasma membranes and intracellular organelle membranes with high levels of PUFA are potential aldehyde formation sites [3,6,7]. Under stress conditions, the levels of aldehydes sourced from lipid peroxide are significantly increased due to the over-accumulation of reactive oxygen species (ROS). Compared to ROS, aldehydes have a longer half-life and are more stable and deleterious to organisms [3]. Thus, it is not surprising that aldehydes were initially regarded as toxic molecules in biological systems. The amount and activity of aldehydes are determined by the species of PUFA and their corresponding oxidation pathways, which result in a variety of aldehydes with different carbon chain lengths and distinct extents of unsaturation [8,9,10]. Among them, *α*,*β*-unsaturated aldehydes, with aldehyde groups adjacent to C-C double bonds (Figure 1), such as acrolein, (*E*)-4-hydroxy-2-nonenal (HNE), and (*E*)-4-hydroxy-2-hexenal (HHE), are relatively electrophilic. *α*,*β*-unsaturated aldehydes can react with nucleophilic targets, including lipids, proteins and nucleic acids [8], consequently impairing their physiological and biological functions. Following the considerable research attention that these aldehydes have garnered within animal systems, more studies on the occurrence and functions of lipid-peroxide-derived aldehydes in plants are being conducted.

Lipid peroxide-derived aldehydes, which are causally involved in pathophysiological effects in cells, were initially characterized as harmful compounds and referred to as “second toxic messengers” [3,12]. Extensive research revealed that aldehydes trigger cell injury and even cause cell death in animals [13,14]. Moreover, considerable studies demonstrated that the accumulation of aldehydes is linked to human diseases, including Alzheimer’s disease, Parkinson’s disease, atherosclerosis, and liver disease [3,4,15,16,17,18]. The extensive progress in the biosynthesis and function of aldehydes in animals provides inspiration and valuable information for investigating their occurrence and actions in plants [16,19,20,21]. Increasing studies have observed a positive correlation between the level of aldehydes and cellular injury in plants under abiotic stresses, such as heat, chilling, salt and heavy metal stresses [10,22,23,24,25,26]. Although excess accumulation of aldehydes leads to cell damage, recent compelling evidence suggests that lower levels of aldehydes might play a pivotal role in signaling processes in order to regulate plant growth and stress responses [27,28]. The distinct species and dynamic action of lipid peroxide-derived aldehydes in plant cells might be responsible for their detrimental effects or signaling functions in critical cellular processes. In this review, we mainly focus on the generation pathways and biochemical properties of lipid peroxide-derived aldehydes and the detrimental effects of aldehydes under adverse conditions. We also highlight the importance of moderating the levels of lipid peroxide-derived aldehydes that may act as potential signaling molecules in the regulation of plant responses to stress conditions.

## 2. Lipid Hydroperoxide Biosynthesis and Aldehydes Formation

An improved understanding of the biosynthesis pathways of aldehydes in plant cells would facilitate characterizing their physiological and biological functions. Currently, it is clear that aldehydes mainly stem from the cleavage of lipids through the radical-induced peroxidation process [29,30]. The PUFA in lipids are commonly and easily oxidized by ROS, resulting in the formation of LOOH [31]. The resultant LOOH is unstable and frequently fragmented by either non-enzymatic or enzymatic routes [7], leading to the formation of a variety of aldehydes with different carbon chain lengths and unsaturation levels [32,33]. Given that PUFA are principal structural components in the plasma membrane and intracellular organelle membranes, it is evident that these subcellular fractions are the major sites and sources of LOOH and aldehydes [7,22]. Genetic studies have successfully provided partial evidence for the amount and composition of aldehydes. For example, Mano et al. reported that Arabidopsis fad7fad8 mutants, which were unable to convert dienic fatty acids to trienoic fatty acids due to the deficiency of fatty acid desaturases localized in the plastid, contained low levels of aldehydes, such as malondialdehyde (MDA), acrolein, (*E*)-2-pentenal and n-hexanal [10]. To date, more than 200 kinds of aldehyde have been identified in living organisms [29,34], but only a dozen aldehydes have been investigated in plants [6,10,26,35].

The content of aldehydes in plants varies significantly depending on the plant species and growth conditions. By reversed-phase liquid chromatography, Yin et al. (2010) separated and identified 18 aldehydes in tobacco roots. Formaldehyde (50 nmol g^−1^ FW) was the most abundant in wild-type tobacco under normal growth conditions, followed by malondialdehyde and n-heptanal (2–4 nmol FW^−1^), and *α*,*β*-unsaturated aldehydes such as HNE, acrolein, and HHE were present at about 1 nmol FW^−1^. The contents of these aldehydes increased by 50% (octanal) to 540% [(*Z*)-3-hexenal] under aluminum (Al) stress [26]. Mano et al. (2014) determined 16 aldehydes in Arabidopsis leaves by using a liquid chromatography-Fourier transform ion cyclotron resonance mass spectrometer (LC-FTICR-MS) and found that the most abundant in the wild-type was formaldehyde at up to 60 nmol FW^−1^. The content of (*E*)-pentenal was about 20 nmol g^−1^ FW. The contents of the other 14 aldehydes, such as acetaldehyde, malondialdehyde, acrolein, and propanal, were below 8 nmol FW^−1^. In addition, HHE was the least abundant. While in the mutants which lacked the biosynthesis of trienoic fatty acids, the amounts of malondialdehyde, acrolein and (*E*)-2-pentenal were significantly lower [10]. According to the study of Liang et al. (2021), the contents of acrolein, butyraldehyde, (*E*)-2-hexenal (HE), and heptanal increased significantly in the root tips of Al-sensitive wheat genotype after 6 h of Al stress compared with those of the Al-tolerant wheat genotype, and the contents of these aldehydes increased with the duration of Al treatment. The contents of acetaldehyde, isovaleraldehyde, valeraldehyde, HE, heptanal and nonanal were all more than 10 nmol g^−1^ FW and significantly higher than other aldehyde species after 24 h of Al stress. Acrolein and HE were 1.94 and 2.63 times higher in the Al-sensitive wheat genotype than in the Al-tolerant wheat genotype, respectively [35].

### 2.1. Non-Enzymatic Lipid Peroxidation Pathways and Aldehyde Generation

Based on a number of studies, the prominent sources of aldehydes in plants are derived from non-enzymatic lipid peroxidation, which is a ubiquitous event during oxidative stress. In this way, radical oxidants, including reactive nitrogen species (RNS) such as peroxynitrite (^•^ONOO), and ROS, such as superoxide radicals (O_2_^•−^) and hydroxyl radicals (^•^OH), attack C-C double bond(s) of PUFA and, consequently, aldehydes are generated as byproducts [4]. RNS and ROS are constantly generated under normal physiological conditions but significantly enhanced under stressful conditions [7,15].

**Figure 2:**
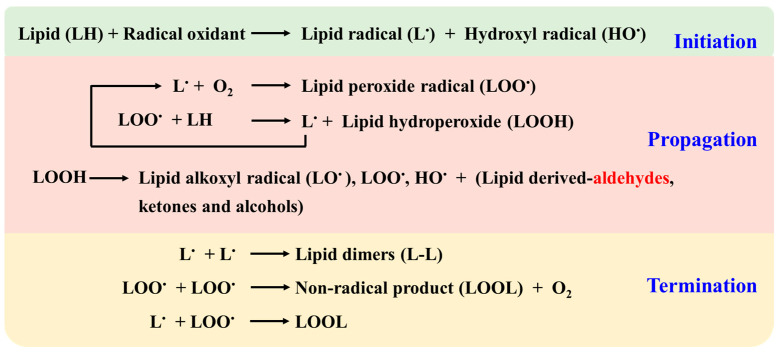
The non-enzymatic pathway of lipid peroxidation and aldehyde generation. This pathway is initiated by radical oxidant and includes three processes: initiation, propagation and termination. Modified from Saxena et al. [11].

Non-enzymatic lipid peroxidation occurs in three major phases: initiation, propagation and termination (Figure 2) [4,7,11,29]. Initiation starts with the abstraction of a bis-allylic hydrogen from a PUFA in a lipid by a radical oxidant, resulting in lipid radical formation. The propagation stage begins with the addition of molecular oxygen to a lipid radical and forms a lipid peroxide radical, which abstracts hydrogen from another PUFA to generate a new L^•^ and an unstable LOOH. LOOH then undergoes Hock cleavage and *β*-scission to generate lipid-derived electrophiles such as aldehydes, among others. According to a study using purified chemical systems, it is estimated that a single initiation step can oxidatively damage 200~400 lipid molecules, roughly equal to 200~400 propagation cycles [36]. The termination of the lipid peroxidation process occurs when two free-radical species react to eliminate the unpaired electrons. Hence, the level of aldehydes is strictly correlated to radical oxidants and the subsequent oxidative stress conditions of PUFA.

### 2.2. Enzymatic Generation Pathways of Lipid-Derived Aldehyde

**Figure 3:**
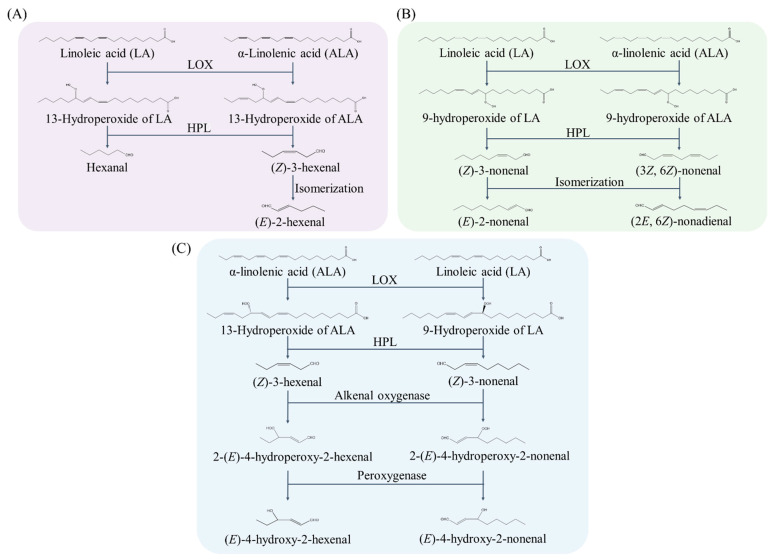
The enzymatic pathway of aldehydes formation from linoleic acid and *α*-linolenic acid. Here, we take several representative aldehydes as examples. LOX, lipoxygenase; HPL, hydroperoxide lyase. (**A**,**B**) were modified from Vincenti et al. [5]. (**C**) was mmodified from Takamura et al. [37].

Regarding the enzymatic pathway contributing to aldehyde generation, several lines of evidence demonstrate that some unique lipid-derived aldehydes are generated through enzymatic catalysis [37,38,39,40]. The most well-known aldehydes sourced from the enzymatic reduction of fatty acids are the C6 and C9 aldehydes. On this route, C6 and C9 aldehydes are usually formed from α-linolenic acids (ALA, 18:3, *ω*-3 fatty acid) and linoleic acids (LA, 18:2, *ω*-6 fatty acid) catalyzed by lipoxygenases (LOX). LOX first catalyze ALA or LA to form 9- or 13-PUFA hydroperoxides, followed by the cleavage of the C-C backbone of the PUFA hydroperoxides catalyzed by hydroperoxide lyase (HPL), a kind of plant cytochrome P450 enzyme. HPL regulates the formation of C6 or C9 volatile aldehydes, such as (*Z*)-3-nonenal, (3*Z*, 6*Z*)-nonadienal, hexanal, and (*Z*)-3-hexenal (Figure 3A,B) [5,41]. Due to the interesting flavoring properties, C6 and C9 aldehydes are also commonly used in the flavor and fragrance industry, cosmetics, and food technology [5,42,43].

Among the various *α*,*β*-unsaturated aldehydes derived from enzymatic routes in plant cells, the pathways for HHE and HNE synthesis have been extensively studied due to their high abundance and multiple biological functions [37,38,39]. It has been unequivocally demonstrated that the biosynthesis of HHE and HNE from ALA and LA is catalyzed by LOX in soybean (*Glycine max* L.) seeds [37]. The LOX-catalyzed 13-hydroperoxylinolenic acid and 9-hydroperoxylinoleic acid were converted into (*Z*)-3-hexenal (HEX) and (*Z*)-3-nonenal (NON) by HPL, respectively. The resultant HEX and NON were converted into 2-(*E*)-4-hydroperoxy-2-hexenal (HPHE) and 2-(*E*)-4-hydroperoxy-2-nonenal (HPNE) by an alkenal oxygenase, followed by the conversion of HPHE into HHE and HPNE into HNE by hydroperoxide-dependent peroxygenase [37,40] (Figure 3C). Mano et al. suggested that (*E*)-2-pentenal was produced from 13-LOOH (an oxidation product of linolenic acid by LOX) via *β*-scission and isomerization [10].

## 3. Aldehydes Induce Cell Injury in Plants

In humans and animals, the potential adverse impacts of aldehydes, especially *α*,*β*-unsaturated aldehydes, on various cells are well-established [17,19,20,44,45]. Lipid-peroxide-derived aldehydes can react with distinct amino acid residues, leading to a Schiff-base through dehydration, and the decoration of amino residues by aldehydes is thought to be irreversible [6,9]. Moreover, unsaturated aldehydes, such as *α*,*β*-unsaturated aldehydes, in which *β*-carbon is positively charged, can react with nucleophilic groups (e.g., histidine-, lysine- and cystine-residues of proteins) or the guanine base of nucleic acids to form a covalent bond by Michael addition. The resulting products from Michael addition can further irreversibly react with other nucleophilic groups to form protein–protein, DNA–DNA and protein–DNA cross links [4,6]. Different from the formation of Schiff base in proteins, Michael addition adds carbonyl moieties (-C=O) to protein molecules to form protein carbonyls (PC). PC is widely considered as the expression of oxidative damage to proteins in plants [6,46,47]. At present, all the adduct products formed via aldehydes reacting with cellular constituents are collectively called advanced lipoxidation end products. Large amounts of enzymes in plant cells are demonstrated to be inactivated as a consequence of aldehydes-based post-translational modifications. For instance, HNE modified the decarboxylating dehydrogenases in the Arabidopsis mitochondria matrix, thus disturbing plant respiratory functions [48]. Yamauchi and Sugimoto found that the MDA-caused modification of the oxygen-evolving-complex 33-kDa protein influenced its binding to the PSII complex and caused the inactivation of the oxygen-evolving complex [49]. In cucumber seedlings, abiotic-stress-induced reactive aldehydes species such as MDA significantly increased the PC content in plant tissues, consequently inhibiting the antioxidant enzymes [47]. However, it must be noted that lipid-derived aldehydes with different carbon chain length and unsaturation levels likely target different protein candidates and perform specific cellular functions [1,9,28]. The potent phytotoxicity of lipid-peroxide-derived aldehydes has been confirmed by exogenous application (Table 1). It is generally agreed that unsaturated aldehydes are more electrophilic, reactive and phytotoxic to plant cells than saturated aldehydes [9,50]. HNE was considered as the most cytotoxic aldehydes among membrane lipid peroxidation products caused by ROS, and its reactivity was 10 times that of malondialdehyde [48]. Acrolein was 400 times more toxic to lettuce seed germination than formaldehyde [9]. Reynolds (1977) compared the toxicity of 14 aldehydes to lettuce seeds and found that the IC50 concentration of unsaturated aldehyde acrolein inhibiting seed germination was 0.043 mM, while the saturated aldehyde propionaldehyde concentration was 13.7 mM [51]. Mano et al. (2009) compared the effects of several short chain reactive aldehydes (C1-9) on chloroplast photosynthesis and found that the inhibition of unsaturated aldehydes on CO_2_ photoreduction was generally higher than that of saturated aldehydes, and the inhibition of acrolein was the strongest, followed by HNE [52]. However, even unsaturated aldehyde had different physiological effects on plants. Compared with (*E*) 2-hexenal, HNE inhibited tobacco root elongation at a lower concentration, indicating that HNE was more toxic to plants than (*E*) 2-hexenal [26]. Taken together, these results suggest that the toxicity of aldehydes depends on species and concentrations and relies on the species and growth conditions of plants.

## 4. Endogenous Aldehyde Levels in Plants under Abiotic Stress Conditions

**Figure 4:**
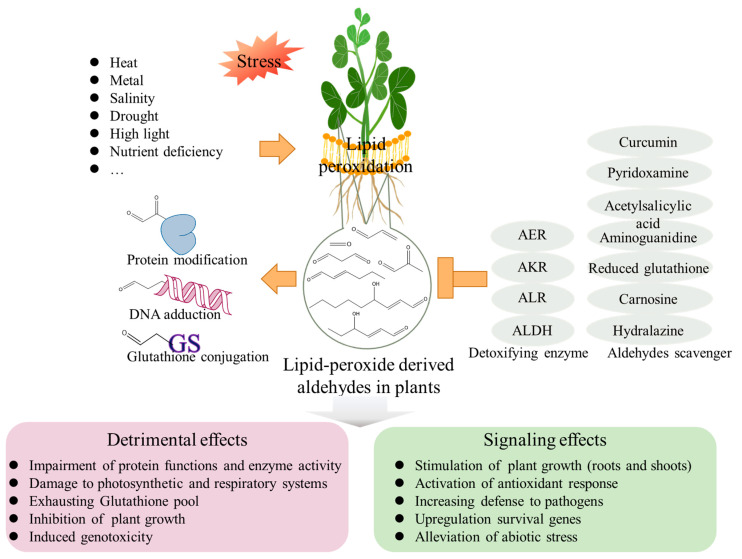
The role of lipid-peroxide-derived aldehydes in plants. Lipid-derived aldehydes play a dual role in plant biology. High concentrations of reactive aldehydes could react with cellular macromolecular compounds, such as proteins, DNA and glutathione, resulting in poor plant growth. Aldehydes also have signaling effects in plants. Certain aldehydes at appropriate concentrations could upregulate survival genes, activate antioxidant responses, increase the defense to pathogens and even stimulate plant growth. In addition, several enzymes and small-molecule compounds with detoxifying capacities of aldehydes can promote plant growth: AER, 2-alkenal reductase; AKR, aldo–keto reductase; ALR, aldose/aldehyde reductase; ALDH, aldehyde dehydrogenase.

Many studies concerned about the involvement of aldehydes in plant responses to abiotic stress have been conducted during the past two decades, and a variety of articles on this topic have been published [9,26,50,64,65] (Table 1). In plants, aldehydes usually remain at a constant level under normal growth conditions. However, their concentrations are strongly affected under unfavorable conditions, such as heat, ultraviolet-B irradiation, nutrient deficiency, and salt stress [14,22,66,67,68]. To remove aldehydes and the subsequent protection from aldehyde-induced injury, plants have adopted several systems to detoxify these toxic compounds. A plethora of enzymes and antioxidant biological molecules detoxifying aldehydes have been discovered and characterized in plants (Figure 4). Transgenic plants overexpressing aldehyde-detoxifying enzymes decreased aldehyde levels and enhanced tolerance to stress conditions [23,69,70,71,72,73]. Moreover, small molecules, such as acetylsalicylic acid, aminoguanidine, carnosine, curcumin, hydralazine, reduced glutathione (GSH), and pyridoxamine non-enzymatically demonstrated a detoxification of aldehydes and rescued plant growth [23,47,50,74].

Heat stress causes a reduction in leaf photosynthesis, which in turn causes crop production losses. With the onset of global warming, heat stress is becoming a more frequent event and, consequently, a critical limiting factor to sustainable agriculture [75]. Several studies investigated the association between aldehydes and heat stress. Chloroplasts are the sites of photosynthesis, as well as the major organelles where MDA are generated. Through in vitro experiments using purified protein (BSA and Rubisco), Yamauchi et al. showed that MDA triggered by a high temperature and oxidative stress caused protein modification, leading to a loss of Rubisco activity [22]. In heat-stressed spinach and Arabidopsis plants, specific proteins modified by MDA were also detected [22]. All of these results indicate that lipid-derived aldehydes are crucial phytotoxic components of heat stress. Fortunately, previous studies found that various genes and enzymes related to aldehyde detoxification were strongly induced by heat stress in plants, especially more tolerant species [76,77,78]. Transgenic tobacco, which overexpressed rice aldo-keto reductase (AKR) and reduced aldehyde to alcohol, exhibited a higher AKR activity, and presented lower levels of MDA and a higher resistance to high temperatures than the wild-type plants [77].

Drought stress negatively affects various biological processes of plants and ultimately decreases crop productivity [79]. Among other consequences, drought causes a rapid and excessive accumulation of ROS, which leads to a lipid peroxidation chain reaction causing an increase in aldehydes [62,80,81]. The scavenging of aldehydes confers plants’ tolerance to drought stress. For example, it was shown that abscisic acid application significantly decreased the level of H_2_O_2_ and MDA and increased tolerance to drought stress in wheat seedlings [82]. The ectopic synthesis of aldose/aldehyde reductase (ALR) in tobacco plants reduced the production of MDA and conferred tolerance against drought stress [68]. Hideg et al. also found that transgenic tobacco with high expression of ALR appeared more tolerant to UV-B stress following drought than wild plants [66]. In addition, tobacco and Arabidopsis transgenic lines overexpressing the soybean antiquitin-like ALDH7 enzyme displayed a lower concentration of reactive aldehydes and enhanced tolerance to drought, salinity, and ROS-producing chemical treatments [80]. The increased activities of Syntrichia caninervis ALDH21 in transgenic tobacco also appeared to constitute a detoxification mechanism that limits aldehyde accumulation and oxidative stress in plants under drought stress [73].

Light is a primary source of energy for green plants. However, a high-light intensity decreases the rate of photosynthesis [83]. High-light stress causes photosystem II to generate excessive ROS that can react with membrane lipids, producing reactive aldehydes [84,85]. In leaves of wild-type tobacco, 2-alkenals, such as acrolein, (*E*)-2-pentenal, and (*E*)-2-hexenal, were increased by 70–290% after 30 min high-light illumination [58]. It is well-known that aldehydes inactivate CO_2_ photoreduction and inhibit the photosynthesis of plants by depleting GSH in chloroplasts. Thus, the accumulation of aldehydes in chloroplasts irreversibly inactivates multiple enzymes in the Calvin cycle, including phosphoribulokinase, glyceraldehyde-3-phosphate dehydrogenase, fructose-1,6-bisphophatase, sedoheptulose-1,7-bisphosphatase, aldolase, and Rubisco [52]. Transgenic tobaccos with a novel enzyme NADPH:2-alkenal reductase (AER) found in Arabidopsis showed resistance to intense light. Unlike AKR, AER protects leaf cells from photooxidative injury by catalyzing the hydrogenation of the *α*,*β*-unsaturated bond of the photoproduced reactive aldehydes, rather than reducing the aldehyde group [58,86].

Salinity stress represents another serious threat to agriculture. More than 20% of cultivated land worldwide is affected by salt stress, leading to enormous economic losses [87,88]. Salinity enhances the formation of ROS in plants. Acting downstream of ROS, aldehydes such as MDA significantly accumulate under salt stress, affecting the biological functions of lipids and proteins and inhibiting plant growth and productivity [59]. In Arabidopsis, several aldehydes such as HNE increase in leaves with the presence of salt stress treatment. Furthermore, immunoblotting using distinct aldehyde antibodies revealed that proteins undergo specific modifications by different aldehydes, such as HNE, HHE, MDA, acrolein and crotonaldehyde [46], while the overexpression of AKR contributes to the detoxification of these cytotoxic aldehydes and confers tolerance to salinity stress in tobacco and barley [59,64]. Recent studies have shown that aldehyde dehydrogenase (ALDH) is also important in the resistance to salt stress in plants [34,89]. The expression of genes encoding the plant ALDH proteins, converting aldehydes into corresponding carboxylic acids, can be upregulated by salt stress, and thus improve plant adaption to salinity [34,71,72,90,91,92].

Nutrient deficiency directly restricts crop yield and quality, a common problem in agricultural production [93]. Increased ROS accumulation and oxidative damage have been frequently observed in different plant tissues following mineral nutrient imbalance [94,95,96,97]. For example, plants with low nitrogen levels experience serious lipid peroxidation and accumulate high levels of lipid-derived reactive aldehydes. The upregulation of AER expression enhanced the tolerance of maize to low nitrogen levels by alleviating oxidative stress and improving nutrient use efficiency [14]. Magnesium deficiency is one of the most prevalent physiological disorders, causing crop yield and quality reduction. In citrus plants, it has been shown that magnesium deficiency induced lipid peroxidation and increased the accumulation of MDA in leaves and roots [61]. Magnesium deficiency also caused MDA elevation in the leaves of maize and rice [98,99]. Much attention has been focused on the functions of ROS in regulating plant responses to nutrient deficiency. However, there is a lack of knowledge on aldehydes’ participation in plants exposed to mineral nutrient imbalance.

Metal pollutants are tremendous threats to the ecosystem. Metal toxicity is one of the significant abiotic stresses and has hazardous effects on plants [88,100,101,102]. A common consequence of metal stress is ROS accumulation, which accounts for the formation of toxic aldehydes [26,35,64]. Under Al stress, more than a dozen aldehydes were characterized in tobacco and wheat roots [26,35,63]. Aldehydes are proposed as the major factor suppressing root growth induced by Al stress. It was further confirmed by the fact that removal of 2-alkenals from the tissue through the overexpression of AER reduces the symptoms of Al toxicity [26]. In addition, heterologous expression of the AKR enzyme in barley plants elevated the tolerance to reactive aldehyde and countered the deleterious effects of cadmium (Cd)-induced oxidative stress [64]. The overexpressed transcription of *Ath-ALDH3* in Arabidopsis thaliana limited the accumulation of lipid-peroxide-derived reactive aldehydes MDA and improved plant tolerance to heavy metals Cu and Cd [71]. Apart from the above-mentioned detoxification systems, other compounds such as GSH and carnosine are also crucial for aldehyde removal under stress conditions. It was found that a high level of GSH contributes to the detoxification of these highly toxic aldehydes in transgenic Arabidopsis overexpressing glutathione reductase under Al stress [23]. The scavenging of lipid-derived aldehydes by carnosine significantly reduced Al accumulation in root tips of wheat [63].

Under all of these stresses mentioned above, there is a close relationship between aldehydes and cell damage. Aldehydes are becoming useful markers for evaluating plant status under abiotic stresses [32].

## 5. Signaling Effects of Aldehydes on Plants

Despite considerable research, these reactive compounds are labeled as toxic messengers. Recent evidence demonstrates that lipid-derived aldehydes play important signaling roles in plant growth and stress responses [29,31] (Table 2).

Aldehydes have dual biological effects. Aldehydes adversely affect normal plant growth at high concentrations. However, certain aldehydes can stimulate plant growth and elicit appropriate responses such as regulation of gene expression at appropriate concentrations. Exposure of *Arabidopsis thaliana* to low levels of MDA results in the upregulation of various genes related to abiotic/environmental stress, such as oxidative-stress-, drought-stress-, and heat-shock-related genes [27]. Yalcinkaya et al. found that low concentrations of three kinds of aldehydes (HNE, HHE and acrolein) stimulated *Arabidopsis* growth under non-stress conditions by elevating the activities of H_2_O_2_ scavenging enzymes and downregulating ROS signaling mediated by NADPH oxidase [105]. Low levels of exogenous HNE, HHE and acrolein increased the root length and fresh weight of halophytic model plant *Eutrema parvulum* under salt stress and might be acting as a downstream signal to elevate the activities of H_2_O_2_ scavenging enzymes and regulate ion homeostasis [105].

When plants sustain physical damage or disease, the signature of PUFA derivatives changes, which is associated with plant response and defense processes. The PUFA derivatives simultaneously produced during attacks might help neighboring healthy cells perceive the presence of dead or dying cells, thus activating cellular protection systems, such as *GST* gene expression [104]. It is worth noting that MDA and other reactive *α*,*β*-unsaturated carbonyls are likely responsible for *GST1* activation [104]. It has been suggested that C6 and C9 aldehydes could act as important signaling molecules that participate in triggering defense responses during plant–pathogen interactions [110,111]. Evidence shows that three-week-old *Arabidopsis* seedlings treated with 1 mmol/L vaporized (*E*)-2-hexenal showed a strong resistance to a necrotrophic fungal pathogen *Botrytis cinerea* [106].

Programmed cell death (PCD) is a form of active and genetically controlled progress. As an important strategy to eliminate specific cells under developmental or environmental stimuli, PCD has been considered an adaptive mechanism for plants under unfavorable conditions [112]. Biswas and Mano (2015) found that aldehydes as mediators are just downstream of ROS for initiating PCD [50]. The transgenic tobacco overproduction of AER suffered less PCD in root epidermis after salt treatment due to lower aldehyde levels [50]. They proposed that unsaturated aldehyde was more likely to induce PCD than saturated aldehyde. For example, exogenous unsaturated aldehyde acrolein and HNE caused PCD of tobacco BY-2 cells at a concentration of 0.2 mM, while exogenous saturated aldehyde propionaldehyde induced PCD when the concentration reaches 50 mM [50]. In addition, a similar conclusion was obtained in tobacco. The exogenous concentration of propionaldehyde triggering PCD in tobacco root hair was several orders of magnitude higher than that of acrolein and HNE [50]. Liang et al. (2023) found that lipid peroxide-derived short-chain aldehydes promoted Al-triggered PCD probably through activating caspase-3-like protease in wheat roots [109].

Senescence is a series of decline processes of plants caused by internal and external factors, resulting in local or over-all death of plants [113]. Aldehydes were also involved in the senescence process of *Arabidopsis* silique. The *Arabidopsis* aldehyde oxidases 4 (AAO4) could efficiently oxidize an array of aldehydes to less toxic acids. Siliques of AAO4-knockout lines accumulated higher levels of MDA and acrolein and showed a premature senescence phenotype under UV-C irradiation and dark stress. Exogenous aldehydes caused silique senescence of AAO4, while wild-type did not, which further proved that aldehydes participated in senescence [56].

Functions of aldehydes in stomatal movement are also being revealed. Islam et al. (2015) found that acrolein inhibited light-induced stomatal opening through inhibition of inward-rectifying potassium channels in guard cells of *Arabidopsis thaliana* [107]. They further demonstrated that acrolein and HNE stimulated stomatal closure as an intermediate downstream of hydrogen peroxide (H_2_O_2_) production in the ABA signaling pathway in guard cells of *Nicotiana tabacum* and *Arabidopsis thaliana* [103]. Murakami et al. (2022) reported that acrolein and HNE achieved strict control of stomatal aperture through inhibition of H^+^-ATPase activation by blue light in addition to Ca^2+^-permeable cation channels activation and K^+^ channel inactivation [108].

Aldehydes were found to play a significant role in reinforcing the auxin signaling for lateral-root formation by facilitating the degradation of Aux/IAA downstream of ROS. Acrolein showed the strongest effects on the promotion of lateral root formation. Saturated aldehydes induced lateral root formation at higher doses than unsaturated aldehydes. In contrast, n-hexanal inhibited lateral root formation [31,57].

## 6. Conclusions and Perspectives

Aldehydes stem from lipids by non-enzymatic or enzymatic processes and are extensively involved in plant physiological processes. Different lipid species result in unique aldehydes with different lengths of carbon chain and levels of unsaturation, which determine their functions and reactivities in plants. Aldehydes can easily modify proteins and nucleic acid, interfering with their properties and functions. Thus, for a long time, lipid-derived aldehydes were regarded as cytotoxins. Therefore, aldehyde levels, to some extent, can reflect the health status of plants. Aldehydes, in particular MDA, are already regarded as a biomarker for the quantitative analysis of lipid peroxidation in several studies [114,115,116,117,118]. Studies using model plants identified several enzymes with the detoxification capacity of aldehydes. However, the specificity and selectivity of these enzymes to aldehydes have not been extensively studied and the detoxification mechanisms in other crop plants have not been fully elucidated. Moreover, a few studies only focused on the intracellular effects of aldehydes. Investigations into whether aldehydes affect extracellular components are limited. Furthermore, a previous report suggested that MDA could potentially downregulate cell wall-related genes [27] and a new study proposed that short-chain aldehydes increase Al retention and sensitivity by enhancing cell wall polysaccharide contents and pectin demethylation in wheat seedlings [63]. Therefore, future studies should aim to comprehensively define the toxic effect of aldehydes, control aldehydes production in a timely manner, and extensively explore the detoxification mechanisms of aldehydes to combat abiotic stresses in plants.

Aldehydes have dual roles. Certain aldehydes, endogenously produced or exogenously applied at appropriate concentrations, play roles in signaling effects and benefit plants, such as through initiating PCD, regulating stomatal movements, activating antioxidant defense, and inducing senescence. However, whether aldehydes form signaling crosstalk with other second messengers is still unclear. Some lipid-derived aldehydes such as C6-aldehydes are responsible for the “grassy beany” odor of soybean, and various aldehydes and other alcohols constitute the “fresh green” flavors of fruits and vegetables [111,119,120]. Therefore, it might be possible to extract certain aldehydes from plants and transform them into valuable products, such as food additives, bioactive supplement agents, pesticides, and fungicides.

## Figures and Tables

**Table 1:** Toxic effects of aldehydes in plant biology.

Growth Condition	Plant Species	Ways of Aldehydes Treatment	Detailed Information	References
Normal condition	Lettuce (*Lactuca saliva* L.)	Exogenous application	14 kinds of aldehydes showed inhibition of germination of Lettuce	[51]
	Potato tuber mitochondria	Exogenous application	HNE inhibited mitochondrial decarboxylating dehydrogenases and inhibited O_2_ consumption	[53]
	*Arabidopsis* cell	Exogenous application	Mitochondrial matrix proteins by HNE resulted in the reduction of oxygen consumption in mitochondria	[48]
	*Arabidopsis thaliana*	Exogenous application	Acrolein decreased the *F_v_*/*F_m_* ratio	[54]
			(*E*)-2-hexenal inhibited root elongation	[55]
			(*E*)-2-hexenal and (*Z*)-3-hexenal decreased the *F_v_*/*F_m_* ratio	[38]
			Benzaldehyde, citral, hexanal, naphthaldehyde, MDA, acrolein, or HNE caused significant tissue damage and enhanced MDA levels	[56]
			High concentrations of acrolein and HNE caused leaf bleaching and high concentrations of (*Z*)-3-hexenal and n-hexanal caused anthocyanin accumulation	[57]
	Tobacco (*Nicotiana tabacum*)	Exogenous application	HNE and (*E*)-2-hexenal inhibited root growth	[26]
	Wheat (*Triticum aestivum* L.)	Exogenous application	(*E*)-2-hexenal inhibited root growth	[35]
Stress condition	Tobacco (*Nicotiana tabacum*)	Intracellular formation	2-alkenals significantly increased after high-light illumination leading to inactivating CO_2_ photoreduction and GSH depletion	[52,58]
			Roots accumulated higher levels of *α*,*β*-unsaturated aldehydes under Al stress	[26]
			MDA significantly accumulated under salt stress	[59]
			AKR1 overexpressing transgenics accumulated a lower level of MDA under glucose, NaCl and methyl viologen-induced oxidative stress, and showed higher seedling growth	[47]
	Spinach thylakoid membrane and *Arabidopsis thaliana*	Exogenous application and intracellular formation	MDA modification proteins in heat-stressed plants leading to a loss of Rubisco activity	[22]
	Spinach (*Spinacia oleracea*)	Exogenous application and intracellular formation	MDA modification of PSII proteins caused the release of oxygen-evolving complex 33 kDa protein from PSII leading to inactivation of the oxygen-evolving complex, which is promoted in heat and oxidative conditions	[49]
	*Arabidopsis thaliana*	Intracellular formation	Methyl viologen treatment caused the inactivation of the photosystems due to enhanced acrolein and crotonaldehyde accumulation	[60]
			HNE, HHE, acrolein, crotonaldehyde and MDA-modified proteins accumulated in leaves under salt stress	[46]
			Siliques of aldehyde oxidase 4-knockout lines accumulated elevated levels of MDA and acrolein, inducing a premature senescence phenotype under UV-C irradiation and dark stress	[56]
	Citrus	Intracellular formation	MDA significantly accumulated in leaves and root with a magnesium-deficiency condition	[61]
	*Labisia pumila* Benth	Intracellular formation	MDA content increased in drought-stressed plants	[62]
	Cucumber	Intracellular formation	MDA accumulation, protein carbonyls content increase under glucose, NaCl and methyl viologen-induced oxidative stress	[47]
	Wheat (*Triticum aestivum* L.)	Intracellular formation	Roots accumulated higher level of short-chain aldehydes under Al stress	[35,63]
		Exogenous application	(*E*)-2-hexenal exacerbated Al accumulation	[63]

**Table 2:** Signaling effects of aldehydes in plant biology.

Plant Species	Signaling Functions	Detailed Information	References
Tobacco BY-2 cell	Initiate programmed cell death (PCD)	Endogenous HNE and acrolein mediating hydrogen peroxide-induced and salt-induced PCD	[50]
		Tobacco cells exposed to HNE and acrolein suffered PCD	[50]
Tobacco	Regulate stomatal movements	Acrolein and HNE mediated methyl jasmonate-induced stomatal closure	[103]
Arabidopsis thaliana	Activate antioxidant defense	Stomatal Closure	[104]
		Exogenous MDA powerfully induced the expression of GST and APX genes	[27]
		Exogenously applied HNE, HHE and acrolein elevated the activities of H_2_O_2_ scavenging enzymes and downregulated NADPH oxidase	[105]
	Activate pathogen defense	Exogenous (*E*)-2-hexenal activated defense genes and induced resistance against a necrotrophic fungal pathogen	[106]
	Deter invaders	Endogenous C6-aldehydes accumulated to deter invaders in disrupted tissues	[38]
	Induce senescence	Siliques of aldehyde oxidase 4-knockout lines accumulated higher levels of MDA and acrolein, inducing a premature senescence phenotype under UV-C irradiation and dark stress	[56]
		Exogenous benzaldehyde, citral, hexanal, naphthaldehyde, MDA, acrolein, or HNE caused senescence symptoms	[56]
	Promote lateral root formation	Reactive oxygen species and reactive aldehydesconstitute a feed-forward loop in auxin signaling for lateral root formation	[57]
	Regulate stomatal movements	Acrolein inhibited light-induced stomatal opening through inhibition of inward-rectifying potassium channels in guard cells	[107]
	Regulate stomatal movements	Acrolein and HNE mediated methyl jasmonate-induced stomatal closure	[103]
	Regulate stomatal movements	Acrolein and HNE inhibited blue-light-dependent activation of the plasma membrane H^+^-ATPase and stomatal opening	[108]
Eutrema parvulum	Activate antioxidant defense	Exogenous HNE, HHE and acrolein increased root length and fresh weight under salt stress and might be acting as a downstream signal to activate H_2_O_2_ scavenging enzymes and regulate ion homeostasis	[105]
Wheat (*Triticum aestivum* L.)	Mediate PCD	Short-chain aldehydes (*E*)-2-hexenal promoted Al-triggered PCD probably through activating caspase-3-like protease in wheat roots	[109]

## Data Availability

Not applicable.
